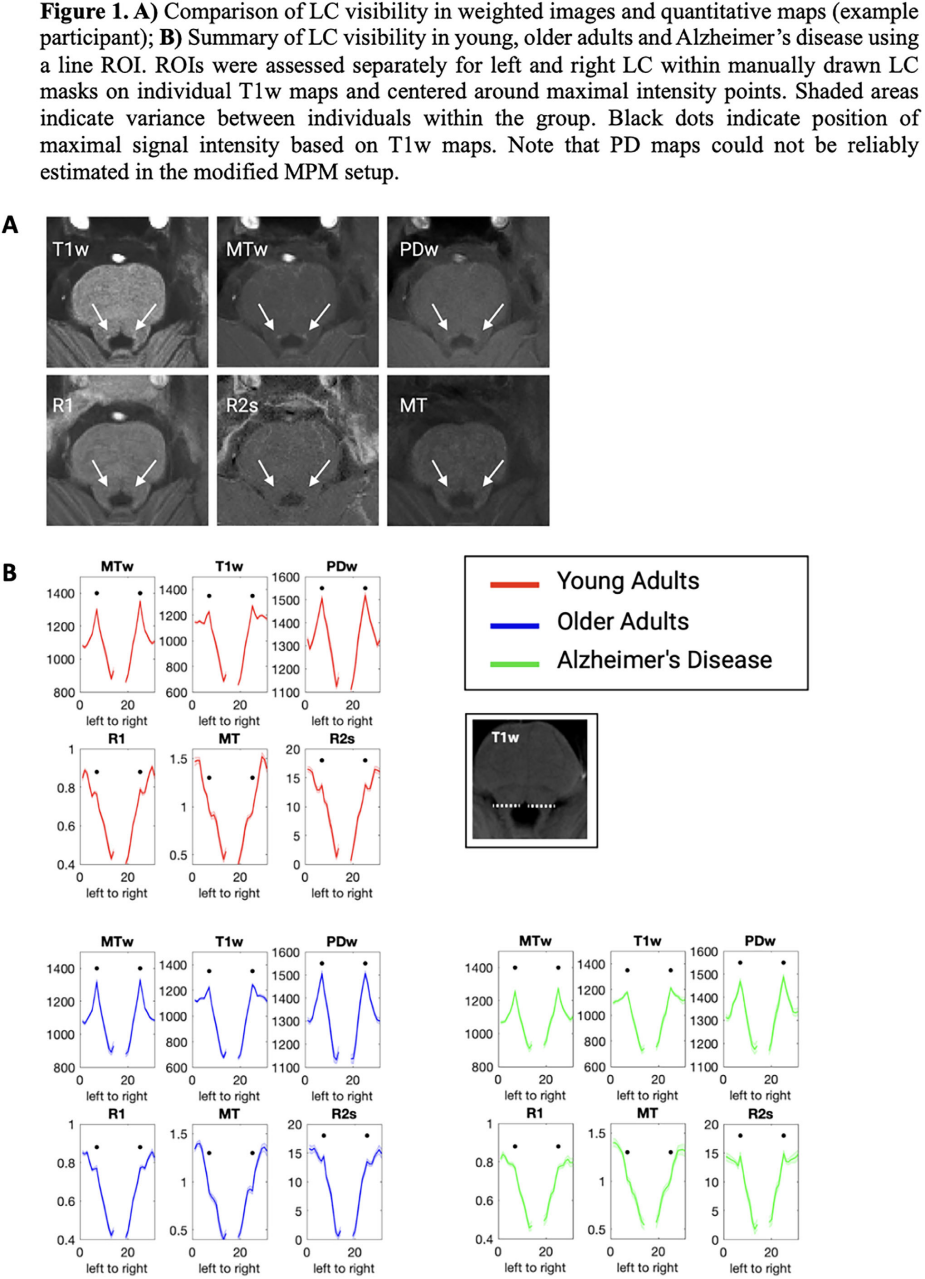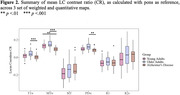# Quantitative multi‐parameter mapping of locus coeruleus in aging and Alzheimer’s disease

**DOI:** 10.1002/alz.095275

**Published:** 2025-01-09

**Authors:** Sabrina Lenzoni, Grazia Daniela Femminella, Clare Loane, Elif Kurt, Millie Duckett, Nikolaus Weiskopf, Martina F Callaghan, Ray Dolan, Robert J Howard, Emrah Düzel, Dorothea Hämmerer

**Affiliations:** ^1^ University of Innsbruck, Innsbruck Austria; ^2^ University of Naples Federico II, Napoli Italy; ^3^ Institute of Cognitive Neuroscience, University College London (UCL), London United Kingdom; ^4^ Aziz Sancar Institute of Experimental Medicine, Istanbul University, Istanbul, Istanbul Turkey; ^5^ Max Planck Institute for Human Cognitive and Brain Sciences, Leipzig Germany; ^6^ Wellcome Centre for Human Neuroimaging, University College London (UCL), Queen Square Institute of Neurology, London United Kingdom; ^7^ Max Planck Centre for Computational Psychiatry and Ageing, University College London, London, London United Kingdom; ^8^ Division of Psychiatry, University College London, London United Kingdom; ^9^ Center for Behavioral Brain Sciences (CBBS), Magdeburg Germany; ^10^ University Hospital Magdeburg, Magdeburg Germany; ^11^ German Center for Neurodegenerative Diseases (DZNE), Magdeburg Germany; ^12^ Institute of Cognitive Neurology and Dementia Research (IKND), Otto‐von‐Guericke University, Magdeburg Germany; ^13^ Department of Neurology, Otto‐von‐Guericke University, Magdeburg Germany; ^14^ Department of Psychiatry and Psychotherapy, Otto‐von‐Guericke University, Magdeburg Germany

## Abstract

**Background:**

The Locus Coeruleus (LC) is prominently affected by neuronal loss in the earliest stages of Alzheimer’s disease (AD). Assessing LC integrity can serve as an important early biomarker for assessing AD progression. Neuromelanin (NM) accumulates in LC neurons and NM imaging has therefore been proposed as a means of imaging the LC. As signal intensity is taken as a proxy for cell density, a quantitative imaging approach of the LC, which is less variable across sites and time is desirable. The present study used a multi‐parameter mapping (MPM) protocol optimized for LC imaging to compare weighted and quantitative maps in healthy younger, healthy older adults and individuals with AD.

**Methods:**

Structural MRI data was acquired in a group of 26 healthy young adults, 26 healthy older adults and 26 individuals with Alzheimer’s disease. Three sets of T1‐weighted, MT‐weighted, and PD‐weighted images yielded quantitative maps (R1, MTsat, PD, and R2*) in each individual within one scan session. Qualitative and quantitative methods were used to assess weighted and quantitative maps for LC imaging across groups.

**Results:**

Qualitatively, LC visibility was higher in weighted images. The LC was also apparent in R1 maps, but less clearly visible in MTsat and R2* maps (Figure 1). LC contrast ratio (with pons as reference), was reduced in Alzheimer’s disease compared to younger adults as detected by MTw scans (*p* = .001) and to older adults as detected by T1w (*p*<.001), MTw (*p*<.001), and PDw scans (*p* = .007). No group differences were detected in quantitative maps, suggesting less sensitivity to pick up typical LC integrity reductions. PD maps could not be reliably estimated in the modified setup of the MPMs.

**Conclusion:**

Although among the quantitative maps LC was most visible in R1 images, our findings indicate that R1 maps capture the LC signal intensity less well as compared to non‐quantitative LC imaging, as suggested by a qualitative assessment of LC visibility and inability to detect known group differences. Further research should improve sensitivity of quantitative maps for LC assessment by combining sequences capturing different aspects of LC tissue properties.